# Central Macular Thickness in Children with Myopia, Emmetropia, and Hyperopia: An Optical Coherence Tomography Study

**DOI:** 10.1155/2015/847694

**Published:** 2015-06-08

**Authors:** Gordon S. K. Yau, Jacky W. Y. Lee, Tiffany T. Y. Woo, Raymond L. M. Wong, Ian Y. H. Wong

**Affiliations:** ^1^Department of Ophthalmology, Caritas Medical Centre, 111 Wing Hong Street, Kowloon, Hong Kong; ^2^Department of Ophthalmology, University of Hong Kong, Room 301, Level 3 Block B, Cyberport 4, 100 Cyberport Road, Hong Kong; ^3^Department of Ophthalmology and Visual Sciences, Hong Kong Eye Hospital, 147K Argyle Street, Kowloon, Hong Kong

## Abstract

*Purpose.* To investigate the central macular thickness (CMT) in myopic, emmetropic, and hyperopic Chinese children using Optical Coherence Tomography.* Methods. *168 right eyes of Chinese subjects aged 4–18 were divided into 3 groups based on their postcycloplegic spherical equivalent: myopes (<−1.0 D); emmetropes (≥−1.0 to ≤+1.0 D); and hyperopes (>+1.0 D) and the CMT was compared before/after age adjustment. The CMT was correlated with age, axial length, and peripapillary retinal nerve fibre layer (RNFL).* Results.* The mean CMT was 274.9 ± 50.3 *μ*m and the mean population age was 7.6 ± 3.3 years. The CMT was thickest in the myopes (283.3 ± 57.3 *μ*m, *n* = 56), followed by the hyperopes (266.2 ± 55.31 *μ*m, *n* = 60) and then emmetropes (259.8 ± 28.7 *μ*m, *n* = 52) (all *P* < 0.0001). When adjusted for age, myopes had a thicker CMT than the other 2 groups (all *P* < 0.0001) but there was no CMT difference between the emmetropes and hyperopes (*P* > 0.05). There was no significant correlation between CMT with age, axial length, or peripapillary RNFL (all *P* ≥ 0.2).* Conclusion.* Chinese children with myopia had a thicker CMT than those with emmetropia or hyperopia. There was no correlation of the CMT with age, axial length, or peripapillary RNFL thickness.

## 1. Introduction

Macular diseases such as macula edema and macula degenerations (hereditary or acquired) can severely affect central vision. Assessment of macular thickness is essential for the diagnosis and monitoring of these diseases. Optical coherence tomography (OCT) is a noninvasive imaging modality for the objective evaluation of the central retinal morphology as well as the measurement of retinal thicknesses [1, 2]. OCT has been demonstrated to be a well-tolerated investigational tool for glaucoma in children as young as the age of 4 [3]. Previous studies have found that female gender, greater birth weight, and infants with an older gestational age have been associated with a thinner central macular (foveal) thickness (CMT) [4, 5]. The CMT also varies with age and ethnicity [6, 7].

The correlation of refractive errors correlate with CMT is still an area of controversy. Huynh et al. [8] found that the CMT was thicker in hyperopic children while Wakitani et al. [9] reported a thicker CMT in myopic children and Lim et al. [10] found no significant correlation between refractive errors and CMT.

The prevalence of myopia ranges from 22.7% to 38.7% based on large population studies, with a higher prevalence in East Asian regions [11–13]. With age, the refractive status often changes from hyperopia to myopia; hence, it is important to understand the dynamic changes of CMT and refractive errors for the paediatric population. The purpose of this study was to investigate the influence of refractive errors on CMT by comparing its differences in children with myopia, emmetropia, and hyperopia.

## 2. Patients and Methods

The study was conducted in accordance with the Declaration of Helsinki and no patient personal data was disclosed in the study. Study approval was obtained from the Institutional Review Board of the Hospital Authority of Hong Kong. Informed consent was obtained from the parents or legal guardian of the subjects.

This cross-sectional study recruited consecutive cases of paediatric subjects aged 4 to 18, attending the Ophthalmology specialist outpatient clinic of Caritas Medical Centre in Hong Kong Special Administrative Region, from 2013 to 2014. Subjects with only eye, ocular tumors, congenital glaucoma, congenital cataract, congenital nystagmus, microphthalmos, optic nerve or retinal disease, active ocular infections, corneal scars, and severe visual impairment of any cause (Snellen best corrected visual acuity ≤ 0.1) and amblyopia were excluded.

All subjects underwent a complete ophthalmological examination including ocular alignment and motility assessments as well as anterior and posterior segment examinations after pupil dilatation with a Tropicamide 1% and Phenylephrine hydrochloride 2.5% ophthalmic solution (Mydrin-P; Santen Pharmaceutical, Osaka, Japan).

### 2.1. Spherical Equivalent and Axial Length

All subjects received cycloplegic refraction with 3 drops of Cyclopentolate hydrochloride 1% (Bausch & Lomb, 1400 N. Goodman St., Rochester, NY, United States of America) administered 5 minutes apart to relieve all accommodative components. After at least 30 minutes, postcycloplegic autorefraction with a kerato-refractometer (Topcon KR-8900 by Topcon Europe Medical B.V., Essebaan 11, Capelle a/d Ijssel, Netherlands) was performed by an optometrist with at least 5 years of experience with paediatric assessment. The spherical equivalent was calculated in diopters (D). Axial length measurements in millimeters (mm) were obtained with the noncontact optical biometry (IOL Master; Carl Zeiss Meditec AG, Max-Dohrn-Straße 8-10, Berlin, Germany). Axial length measurements were performed 3 times by a single technician who was masked to subjects' clinical information, and the average of the 3 values was recorded. Poor signal values as well as values that differed by more than 0.1 mm were rejected and the measurement was repeated.

### 2.2. Optical Coherence Tomography Imaging

The Spectralis Spectral Domain OCT (Heidelberg Engineering, 1808 Aston Ave., Suite 103, Carlsbad, CA, USA) was performed after cycloplegia, by a single imaging technician who was masked to subjects' clinical information.

### 2.3. Peripapillary RNFL Thickness

Scans were centred on the optic disc with a scanning diameter of 3.5 mm and 768 A-scans were obtained using the high speed (HS) mode. To improve image quality, automatic real time (ART) function was used to obtain multiple frames during scanning and to optimize images by noise reduction. Scans were repeated 3 times and assessed for signal strength and centration. Scans with signal strength quality ≤ 16 or poor centration were excluded. RNFL thickness was analysed with the RNFL Single Exam Report OU with fovea-to-disc technology. The RNFL thickness of each of the 4 quadrants and the global RNFL thickness were recorded in micrometers (*μ*m).

### 2.4. Central Macula Thickness Measurement

The Spectralis OCT has an axial image resolution of 7 *μ*m, a lateral resolution of 14 *μ*m, and a scanning velocity up to 40,000 A scans per second. CMT measurements were acquired using a dense (25-line) horizontal Raster Scan protocol, centred on the fovea with a distance of 240 *μ*m between the horizontal scans. The TruTrack active eye tracking system was used to increase scan quality.

### 2.5. Statistics

Subjects were divided into 3 groups based on their postcycloplegic spherical equivalent: myopic (<−1.0 D); emmetropic (≥−1.0 to ≤+1.0 D); and hyperopic (>+1.0 D). Only the right eye of each subject was used for statistical analysis. Statistical significance was considered when *P* < 0.05. Means were expressed with standard deviations.

One-way ANOVA with Tukey's Multiple Comparison Test was used to compare the CMT among the 3 spherical equivalent groups before and after age adjustment. The* t*-test was used to compare the CMT between female and male subjects.

Pearson correlation was used to analyze the association between the following:

- CMT versus RNFL (global and quadrant) thicknesses,
- CMT versus age,
- CMT versus axial length.

## 3. Results

Of the 168 subjects eligible for the study, the mean age was 7.6 ± 3.3 years. The mean CMT was 274.9 ± 50.3 *μ*m. There were 85 female and 83 male subjects; all were of Chinese ethnicity. There was no difference in the CMT between the female (268.9 ± 52.6 *μ*m) and male (272 ± 45.0 *μ*m) subjects (*P* = 0.7). There were 56 (33.3%) myopic eyes, 52 (31.0%) emmetropic eyes, and 60 (36.7%) hyperopic eyes. The age of the 3 groups was significantly different: 10.1 ± 4.1 years (myopic group), 6.9 ± 2.7 years (emmetropic group), and 6.5 ± 2.1 years (hyperopic group) (all *P* < 0.0001).

There was no statistically significant correlation of the CMT with RNFL, age, or axial length. The parameter means and correlations are summarized in Table 1.

**Figure 1:**
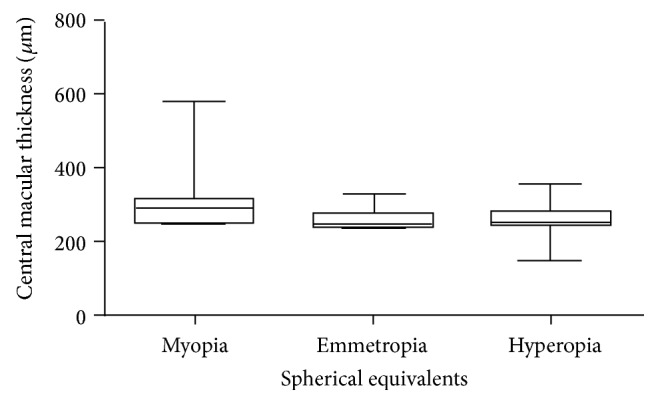
Differences in age-adjusted central macular thickness (mean ± standard deviation) in the myopic, emmetropic, and hyperopic children.

The mean spherical equivalent was −3.9 ± 2.2 D in the myopic group, +0.1 ± 0.5 D in the emmetropic group, and +2.9 ± 1.5 D in the hyperopic group (all *P* < 0.001). The mean CMT in the myopic, emmetropic, and hyperopic groups was 283.3 ± 57.3 *μ*m, 259.8 ± 28.7 *μ*m, and 266.2 ± 55.31 *μ*m, respectively. The CMT in the myopic group was significantly thicker than the emmetropic group (*P* < 0.0001) but there was no significant difference between the mean CMT of the other groups (all *P* > 0.05). Even though age was not found to be associated with CMT, in view of the statistically different age distribution of the 3 spherical equivalent groups, the mean CMT was reanalysed after age matching among the 3 spherical equivalent groups to eliminate the potential influence of age differences. When adjusted for age, the myopic group (298.2 ± 69.6 *μ*m) had a significantly thicker CMT than both the emmetropic (261.3 ± 21.5 *μ*m) and hyperopic group (265.3 ± 40.4) (both *P* < 0.0001). There was no significant difference in CMT between the emmetropic and hyperopic group (*P* > 0.05) (Figure 1).

## 4. Discussion

In our study, 33.3% of the study children had myopia and their mean age (10.1 ± 4.1 years) was significantly older than their emmetropic and hyperopic counterparts (all *P* < 0.0001).

Our findings were in agreement with Fan et al., who likewise reported that, in a population of Chinese children, 36.71 ± 2.87% were myopic and that the prevalence of myopia was positively correlated with age [14]. The higher prevalence of myopia in East Asian Children when compared with European Caucasians was found to be attributed to parental myopia and more myopigenic activities like long hours of near work and few outdoor time [15, 16].

In our study, we found that the mean CMT in the myopic group was 298.2 ± 69.6 *μ*m which was significantly thicker than both the emmetropic (261.3 ± 21.5 *μ*m) and hyperopic group (265.3 ± 40.4 *μ*m) (both *P* < 0.0001). This finding was in agreement with Wakitani et al. [9] and Zhang et al. [6] but in contrast to Huynh et al. [8] who reported that CMT was positively correlated with hyperopia in an Australian paediatric population. These discrepancies can be largely explained by racial and age differences. Huynh et al. [8] only recruited 6-year-olds in their study while our study population consisted of children between the ages of 4–18. Thus, the proportion of hyperopia is much higher in Huynh's population given the younger age and predominant Caucasian race in their study. We are in agreement with the postulation by Wakitani et al. [9] that myopic eyes, the thicker CMT, serves as a compensatory mechanism at the expense of a thinner peripheral retina in order to preserve the fovea, which is more essential to vision.

Previous studies have demonstrated the gender difference of CMT with males who have thicker CMTs [4, 6, 8]. However, we did not defect any difference in CMT between males and females in our study (*P* = 0.7). There was no significant correlation between age and CMT in our study (*r* = −0.09; *P* = 0.26) which was in agreement with Zhang et al., Eriksson et al., and Göbel et al. [6, 17, 18]. Previous studies involving a Hong Kong Chinese population have demonstrated a positive correlation between axial length and CMT as reported by Lam et al. [19] (*r* = 0.374; *P* < 0.001) and Wong et al. [20] (*r* = 5.37; *P* = 0.001). However, we did not find any significant correlation between axial length and CMT in our study (*r* = 0.004; *P* = 0.96). These differences in associations can be attributed to the differences in sample size among different studies as well as differences in scanning protocol used among different OCT machines [21, 22].

Furthermore, there was no significant correlation between the peripapillary RNFL thickness and CMT. To the best of our knowledge, this is first study to investigate this correlation. This serves as a milestone for future research into this area for the paediatric population since, in adults, the peripapillary RNFL thickness has been associated with the CMT [23, 24], which can be used as a proxy measure of the RNFL in glaucoma patients with preexisting anatomical defects of the optic nerve hindering traditional RNFL monitoring by OCT. A longitudinal follow-up of our study population would also be useful to investigate the serial changes in CMT that comes with age and axial length elongation. Nevertheless, this is one of the few studies using the Spectralis OCT machine to quantify the CMT in a Chinese paediatric population and found that children with myopia had a thicker CMT than those with emmetropia or hyperopia. There was no correlation of the CMT with age, axial length, or peripapillary RNFL thickness. As this study was based on a Chinese pediatric population, the results may not be generalizable for other age groups and ethnicities.

## Figures and Tables

**Table 1:** Correlation of CMT with RNFL, age, and axial length.

	Mean	Pearson *r*	*P* value
RNFL			
Inferior	130.9 ± 25.3 *µ*m	0.04	0.64
Superior	126.4 ± 23.1 *µ*m	0.07	0.38
Nasal	65.01 ± 20.2 *µ*m	0.11	0.16
Temporal	88.3 ± 20.5 *µ*m	0.04	0.62

Average	102.7 ± 14.4 *µ*m	0.10	0.20

Age			
7.8 ± 3.5 years	−0.09	0.26

Axial length			
	22.9 ± 1.5 mm	0.004	0.96

RNFL: retinal nerve fibre layer.
